# Mitochondria limit coenzyme Q export under cholesterol biosynthetic stress

**DOI:** 10.1083/jcb.202507174

**Published:** 2026-06-08

**Authors:** Marjana Ndoci, Sharanya Bhattacharya, Ishita Agrawal, Yvonne Hinze, Kathrin Lemke, Anna-Lena Schumacher, Esther Uijttewaal, Ulrich Elling, Steffen Lawo, Patrick Giavalisco, Thomas Langer, Soni Deshwal

**Affiliations:** 1 Metabolism and Cell Death Institute, Molecular Targets and Therapeutics Centre, Helmholtz Zentrum München, Neuherberg, Germany; 2 Max-Planck Institute for Biology of Aging, Cologne, Germany; 3 Institute of Molecular Biotechnology of the Austrian Academy of Science (IMBA), Vienna BioCenter (VBC), Vienna, Austria; 4 ViVerita Discovery, Vienna, Austria

## Abstract

Coenzyme Q (CoQ) is a hydrophobic lipid primarily synthesized in the mitochondria, though it is also present in non-mitochondrial membranes. However, the metabolic pathways that regulate intracellular CoQ distribution are unknown. This study identifies a key role for the mevalonate pathway in regulating CoQ distribution. The mevalonate pathway synthesizes isopentenyl pyrophosphate (IPP) as the precursor metabolite for both CoQ and cholesterol. We show that CoQ synthesis remains stable regardless of whether the mevalonate pathway is upregulated or downregulated. Upregulation of HMG-CoA reductase (HMGCR), indicative of increased mevalonate flux, enhances cholesterol ester synthesis without altering CoQ levels. When the pathway is downregulated, cholesterol synthesis declines, yet mitochondrial CoQ levels are preserved. Under these limiting conditions, mitochondria reduce CoQ export to maintain their internal CoQ pool. While this adaptation sustains mitochondrial respiration, it diminishes extramitochondrial CoQ availability and sensitizes cells to ferroptosis. These findings uncover a mitochondria-driven mechanism that preserves respiratory function by prioritizing CoQ retention during metabolic stress.

## Teaser

Mitochondria prioritize internal CoQ retention when mevalonate flux is compromised, creating a bioenergetic “fortress” to sustain respiration. This strategic reallocation preserves mitochondrial respiration but exhausts extramitochondrial antioxidant defenses, ultimately sensitizing the cell to ferroptotic death.

## Introduction

Coenzyme Q (CoQ or ubiquinone) is a hydrophobic-lipid molecule essential for mitochondrial respiration (Guerra and Pagliarini, 2023; Sood et al., 2025; Baschiera et al., 2021). It functions as a key electron carrier in the electron transport chain (ETC), transferring electrons from complexes I and II to complex III to support oxidative phosphorylation (OXPHOS) (Guerra and Pagliarini, 2023; Martinez-Reyes et al., 2020; Lapuente-Brun et al., 2013). In addition to its role in energy production, CoQ also participates in several mitochondrial metabolic pathways, including pyrimidine biosynthesis, sulfide metabolism, and proline catabolism, by serving as an electron acceptor for enzymes such as dihydroorotate dehydrogenase (DHODH), sulfide-quinone oxidoreductase, and proline dehydrogenase (Awad et al., 2018; Heaton et al., 2020). Because complex III is the only ETC component that can reoxidize CoQ under physiological conditions, mitochondrial CoQ functions as a critical node linking respiration with other metabolic networks (Banerjee et al., 2022).

Disruptions in CoQ biosynthesis or its associated regulatory proteins cause a range of mitochondrial disorders, including Leigh syndrome, underscoring the importance of maintaining CoQ homeostasis (Alcazar-Fabra et al., 2018; Awad et al., 2018; Hargreaves et al., 2020; Montero et al., 2013). Although CoQ is synthesized in the mitochondria, it is also present in extramitochondrial membranes (Guile et al., 2023; Navas et al., 2007). In the plasma membrane (PM), CoQ traps lipid peroxyl radicals, limiting lipid peroxidation and ferroptosis. FSP1, formerly AIFM2, reduces CoQ to ubiquinol, CoQH_2_ in a NAD(P)H-dependent manner (Doll et al., 2019; Bersuker et al., 2019). We previously showed that the mitochondrial rhomboid protease PARL and its substrate STARD7 regulate both CoQ biosynthesis and its intracellular distribution (Deshwal et al., 2023). PARL cleavage regulates the dual localization of STARD7 to the mitochondrial intermembrane space and to the cytosol (Saita et al., 2018). Mitochondrial STARD7 is required for CoQ synthesis, whereas cytosolic STARD7 (cyto-STARD7) facilitates the transfer of CoQ to non-mitochondrial membranes (Deshwal et al., 2023). Overexpression of cyto-STARD7 cells is sufficient to increase CoQ levels in extramitochondrial compartments and confers resistance to ferroptosis, but at the cost of decreased mitochondrial CoQ and impaired respiratory capacity (Deshwal et al., 2023). Conversely, restricting CoQ to mitochondria in the absence of cyto-STARD7 preserves OXPHOS but sensitizes cells to ferroptotic death (Deshwal et al., 2023). These observations suggest that cells must dynamically regulate CoQ distribution to balance respiration with redox protection.

To identify the metabolic pathways that govern CoQ export from mitochondria, we performed a genome-wide CRISPR screen in cyto-STARD7 HeLa cells. This screen revealed that CoQ distribution is influenced by changes in mevalonate pathway activity, which supplies precursors for both CoQ and cholesterol synthesis.

## Results and discussion

### Genome-wide CRISPR knockout screen to identify genes involved in CoQ distribution

**Figure 1.**
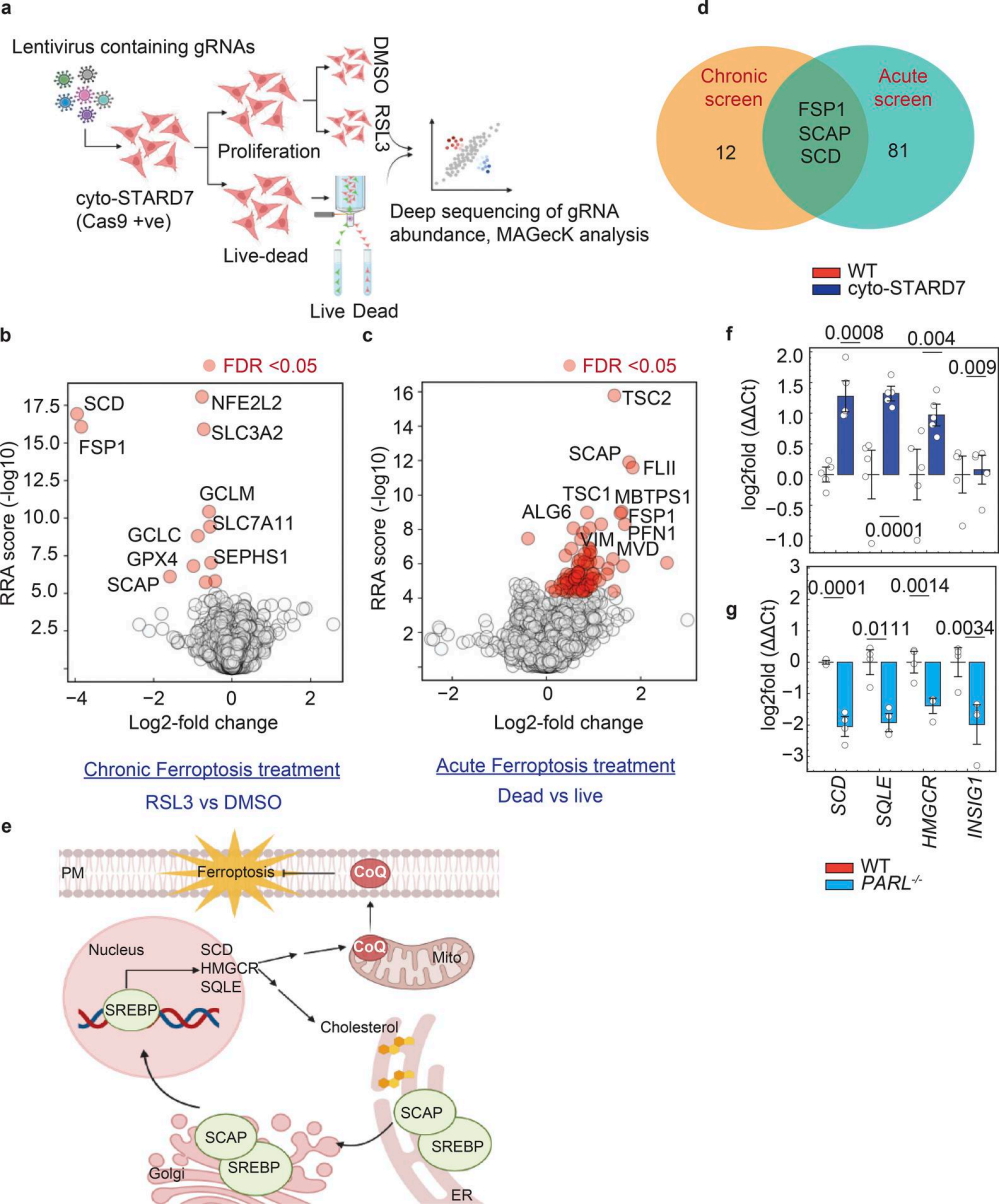
**Genome-wide CRISPR KO screen to identify pathways regulating CoQ distribution. (a)** Ferroptosis-resistant HeLa cells, cyto-STARD7, stably expressing Cas9 were infected with the genome-wide VBC library (each gene targeted by five guide RNAs). The cells were then selected with G418 and subjected to two different types of treatments (*n* = 2 biological replicates). First, for chronic ferroptosis treatment, cells were cultured with DMSO or 100 nM RAS-selective lethal 3 (RSL3) for 2 wk, after which they were collected, and gDNA was isolated for PCR amplification of genome-integrated gRNAs followed by deep sequencing. Second, for acute treatment, cells were treated with 500 nM RSL3 for 48 h. After treatment, cells were trypsinized, stained with a live–dead stain, and sorted into gates indicating live and dead cells. gDNA was isolated from both the live and dead cell pellets, and gRNA abundance was analyzed by deep sequencing. **(b and c)** The x-axis shows gene-level LFCs; the y-axis shows Robust rank aggregation (RRA) scores for b chronic and c acute ferroptosis treatment. Genes with guide RNAs significantly depleted (b) or enriched (c) are highlighted with red circles. The top 10 significant genes are labeled in the Volcano plots. A gene was considered a positive hit if FDR <0.05. **(d)** Venn diagram showing 12 genes with guide RNAs depleted after chronic RSL3 treatment and 81 genes with guide RNAs enriched in the dead cell population after acute RSL3 treatment. Three genes overlap between the two screens: SCD, FSP1, and SCAP. **(e)** Schematic representation showing that both CoQ and cholesterol synthesis are regulated by the SCAP–SREBP pathway. Inhibition of SCAP prevents the recruitment of the SCAP–SREBP complex to the Golgi, inhibiting the transcription of SREBP target genes, including HMGCR, a rate-limiting enzyme in the mevalonate pathway, thereby blocking both CoQ and cholesterol synthesis. The scheme was created using BioRender. **(f and g)** Relative gene expression levels of SREBP-target genes, including SCD, SQLE, HMGCR, and INSIG1, were calculated using the ΔΔCt method with TaqMan probes. HPRT was used as the reference gene. Results were normalized to WT HeLa cells, and the expression of these genes is shown in cyto-STARD7 cells (f) and *PARL*^−/−^ cells (g) (*n* = 4 biological replicates). The error bars represent the 95% confidence interval. P values are shown above the graphs. SQLE, squalene epoxidase; INSIG1, insulin-induced gene 1; PARL, presenilin-associated rhomboid-like protein; GPX4, glutathione peroxidase 4.

Overexpression of cyto-STARD7 confers ferroptotic resistance to cells increasing CoQ export from mitochondria and CoQ localization to the PM (Deshwal et al., 2023). To explore the determinants that regulate CoQ distribution within cells, we performed a genome-wide CRISPR knockout (KO) screen in cyto-STARD7 HeLa cells (Fig. 1 a). We constitutively expressed Cas9 in cyto-STARD7 cells and confirmed Cas9 activity using a BFP-GFP fluorescence reporter (Tzelepis et al., 2016), ensuring efficient gene editing (Fig. 1, a–c). The hypothesis driving this study was that deletion of genes involved in CoQ distribution would modulate the ferroptotic resistance of cyto-STARD7–expressing cells.

We conducted two distinct genome-wide CRISPR screens to capture different aspects of cellular responses to ferroptosis (Michlits et al., 2020). The first screen was a fitness-based assay (Fig. 1 b), in which cyto-STARD7 cells were cultured in the presence of either DMSO or the ferroptosis inducer RSL3 (100 nM) (Bebber et al., 2020) for 2 wk after infection with the gRNA library, establishing a chronic ferroptosis model. This screen aimed to identify cells expressing gRNAs with decreased abundance upon RSL3 treatment, indicating that the corresponding genes contribute to the ferroptosis resistance of these cells. In the second screen, cyto-STARD7 cells were treated with RSL3 at a higher concentration (500 nM) for 48 h, inducing acute ferroptosis (Fig. 1 c). We then used a live–dead stain followed by high-throughput cell sorting, enabling us to separate and analyze both the live cell population and the live–dead stain–positive (membrane-compromised) population. In this screen, we aimed to identify gRNAs that were enriched in the live–dead stain–positive population relative to the live population, as these genes would likely be critical for protecting cells against ferroptosis.

**Figure S1.**
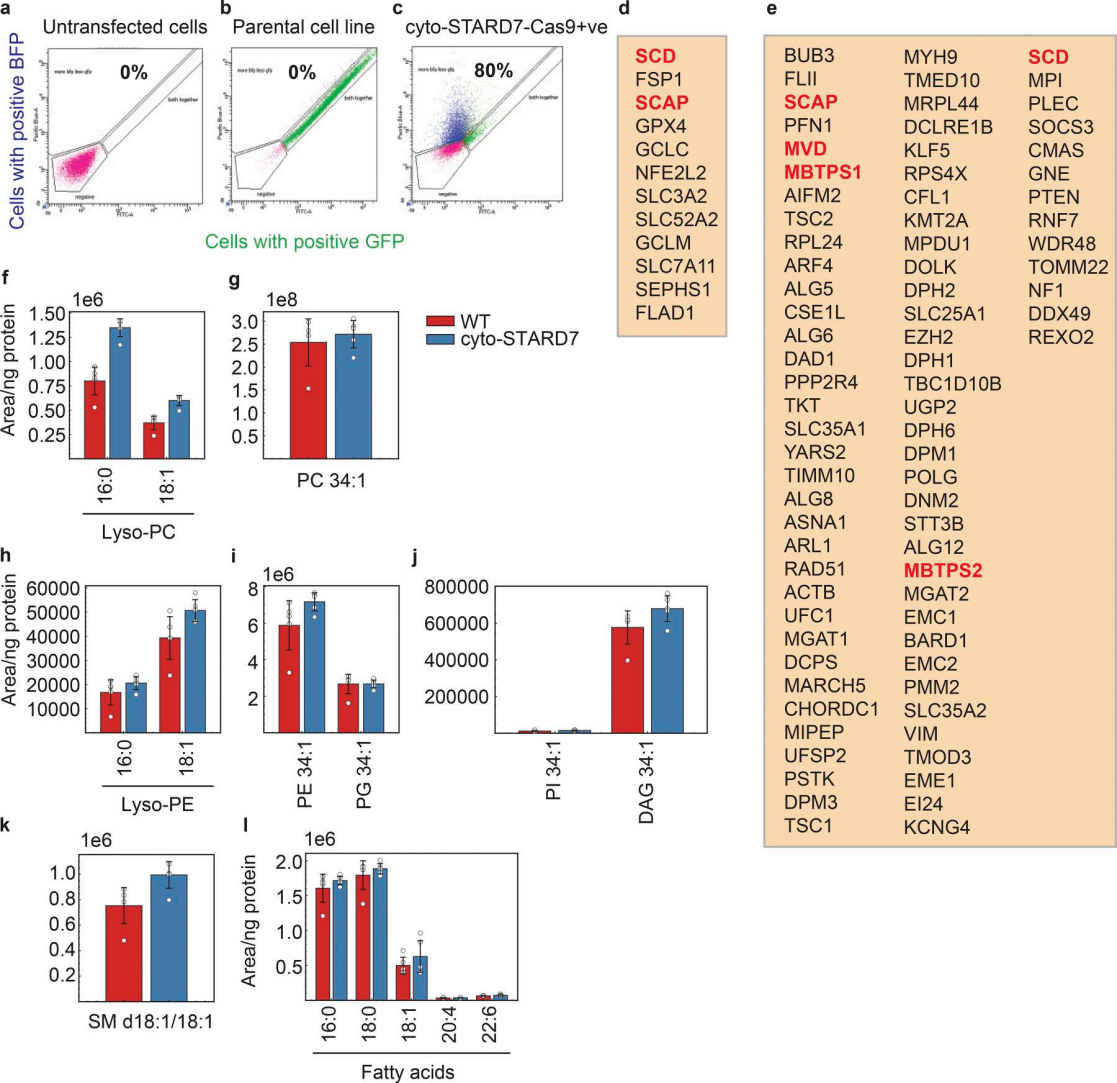
**Functional validation of Cas9 expression and comprehensive lipid profiling in cyto-STARD7 cells. (a–c)** Cyto-STARD7 HeLa cells were transduced with a Cas9-expressing lentivirus and selected with blasticidin to generate a stable, monoclonal population expressing Cas9. To test Cas9 activity, cells were transfected with a BFP-GFP reporter plasmid containing a guide RNA targeting GFP. Green and blue fluorescence were measured in untransfected HeLa cells (a), parental cyto-STARD7 cells lacking Cas9 (b), and cyto-STARD7 cells stably expressing Cas9 (c). In cells without Cas9, both green (GFP) and blue (BFP) signals are visible, indicating no gene editing. In contrast, in Cas9-expressing cells, ∼80% of cells lost green fluorescence but retained blue, demonstrating successful Cas9-mediated GFP KO. **(d and e)** Genes whose guide RNAs are depleted (d) or enriched (e) in chronic and acute ferroptosis screens in cyto-STARD7 HeLa cells. Lists include genes with FDR <0.05: 12 significant genes identified in the chronic screen (d) and 81 in the acute screen (e). Genes highlighted in bold red are part of the SCAP–SREBP and mevalonate pathways. **(f–l)** Levels of various lipid species, including lysophosphatidylcholine (lyso-PC), PC, lysophosphatidylethanolamine (lyso-PE), PE, PG, PI, diacylglycerol (DAG), sphingomyelin (SM), and several fatty acids (saturated: 16:0, 18:0; monounsaturated: 18:1; polyunsaturated: 20:4, 22:6) were measured in WT and cyto-STARD7 HeLa cells using mass spectrometry. Fatty acids were extracted from the polar phase using the methyl-tert-butyl ether (MTBE) method, while all other lipids were extracted from the lipid phase (*n* = 5 biological replicates). Mass spectrometry peak areas were normalized to the protein concentration of the corresponding cell pellets. Error bars represent the 95% confidence interval.

The two screens identified distinct sets of genes, highlighting the importance of the temporal dimension in ferroptosis induction. Notably, FSP1 emerged as one of the top candidates in both screens, confirming its central role in protecting these cells as previously observed (Bersuker et al., 2019; Doll et al., 2019; Deshwal et al., 2023) (Fig. 1, b and c). We next examined all other false discovery rate (FDR)-significant genes identified in both screens, highlighted in red circles (Fig. 1, b and c; and Fig. S1, d and e). In the chronic screen, we detected 12 genes, whose deletion led to reduced fitness of cyto-STARD7 cells treated with RSL3 compared with DMSO. In contrast, the acute screen revealed 81 genes enriched in the RSL3-treated dead cell population compared with live cells, showing that deletion of these genes sensitized cyto-STARD7 cells to ferroptosis within 48 h of treatment (Fig. 1 d; and Fig. S1, d and e). We then searched for common candidates between the two screens. Besides FSP1, SREBP cleavage-activating protein (SCAP) and stearoyl-CoA desaturase (SCD) were common (Fig. 1 d). Both genes are involved in lipid metabolism: SCD synthesizes monounsaturated fatty acids (MUFAs) from saturated fatty acids, while SCAP regulates cholesterol synthesis by binding to SREBP in a cholesterol-dependent manner (Sen et al., 2023; Miyazaki and Ntambi, 2003; Brown et al., 2018).

The SCAP–SREBP pathway is activated when cholesterol levels in the ER are depleted, leading to the translocation of SREBP to the nucleus, where it initiates the transcription of target genes, including 3-hydroxy-3-methylglutaryl-CoA reductase (HMGCR), SCD, insulin-induced gene 1, and squalene epoxidase (Fig. 1 e) (Brown and Goldstein, 1999; Brown et al., 2018). Notably, the acute screen also identified additional components of this pathway, including mevalonate diphosphate decarboxylase, which synthesizes isopentyl pyrophosphate (IPP); membrane-bound transcription factor peptidase, site 1, which encodes the site-1 protease that cleaves SREBP in the Golgi; and membrane-bound transcription factor peptidase, site 2, which encodes the site-2 protease required for the second step of SREBP activation (Fig. S1 e, highlighted in red) (Brown and Goldstein, 1997). Moreover, we observed that targets of the SCAP–SREBP pathway were transcriptionally upregulated in cyto-STARD7 cells and downregulated in cyto-STARD7–deficient cells (i.e., PARL^−/−^ cells) (Fig. 1, f and g), suggesting that the presence of cyto-STARD7 is required to activate SCAP–SREBP pathway.

Taken together, these data suggest that genetic inhibition of the SCAP–SREBP pathway abolishes the protective effect of cyto-STARD7 cells against ferroptosis.

### Cholesterol synthesis is more sensitive to mevalonate pathway inhibition than CoQ synthesis

**Figure 2.**
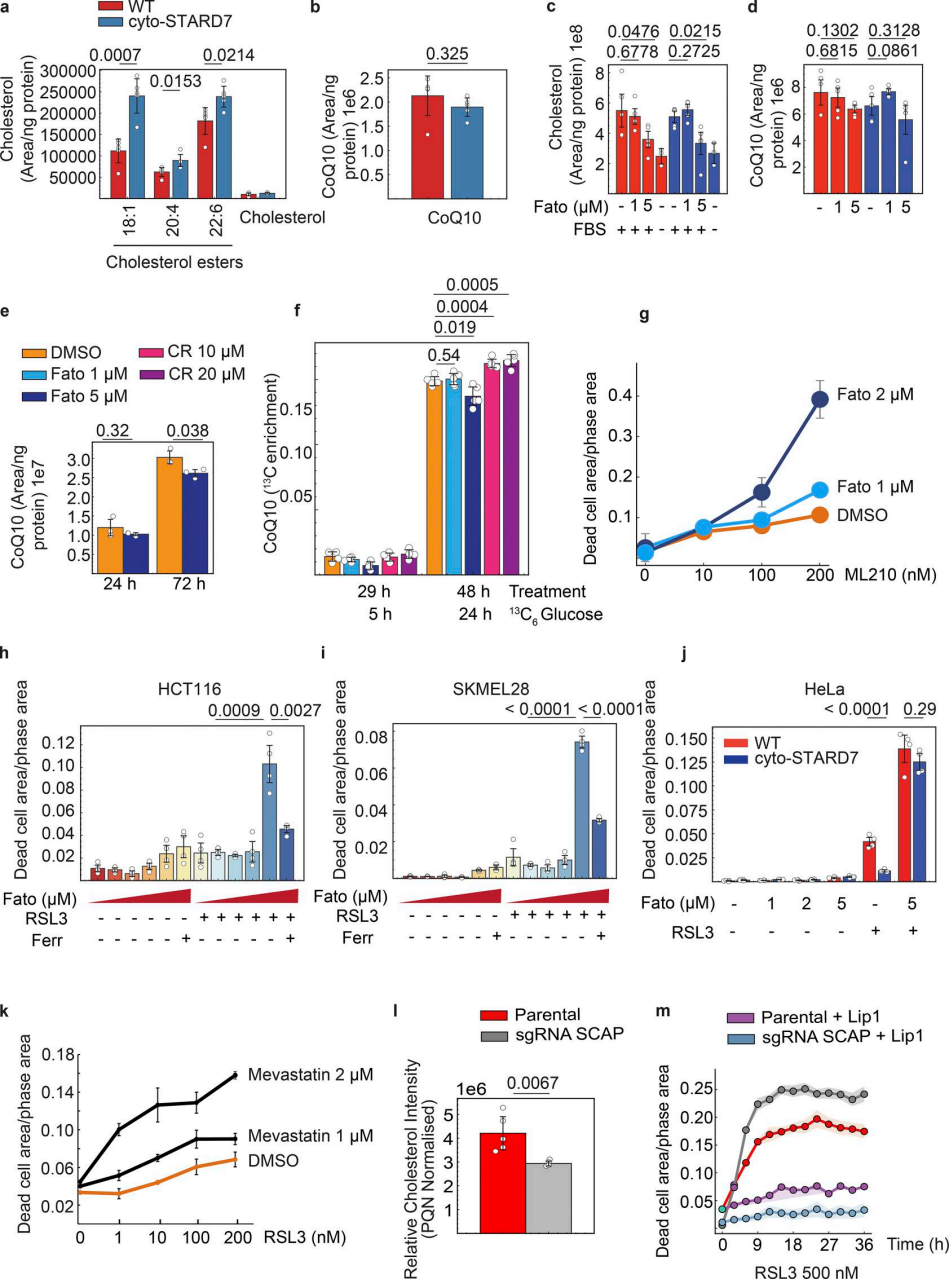
**Kinetic assessment of CoQ and cholesterol upon SCAP activation and inhibition. (a and b)** Cholesterol (a) and CoQ (b) levels measured via mass spectrometry from the lipid phase of WT and cyto-STARD7 HeLa cells (*n* = 5). The peak area is normalized to protein concentration. **(c and d)** HeLa cells were cultured with or without the SCAP inhibitor fatostatin (*n* = 4) at the indicated concentrations. As a positive control for cholesterol measurement, cells cultured in the absence of FBS were used to observe a decline in cholesterol levels (c). The peak areas of cholesterol (c) and CoQ (d) were normalized to protein concentration. **(e)** CoQ levels were measured using mass spectrometry in HeLa cells treated with either DMSO (orange) or the SCAP inhibitor fatostatin (blue) for 24 or 72 h (*n* = 3). The peak area for CoQ was normalized to the protein concentration in the samples. **(f)** HeLa cells were treated with either fatostatin or cholesterol (CR) at the indicated concentrations for 24 and 48 h (*n* = 5). After treatment for 24 h, the culture medium was replaced with DMEM containing ^13^C_6_-labeled glucose. Cells were then snap-frozen either 5 or 24 h after the ^13^C_6_-glucose incubation. To assess newly synthesized CoQ, lipids were extracted from the lipid phase, and ^13^C_6_ enrichment in CoQ molecules was measured using mass spectrometry. **(g–j)** Dose–response analysis of ferroptosis sensitivity in HCT116 colorectal cancer cells (h), SK-MEL-28 melanoma cells (i), and HeLa cells (WT and cyto-STARD7) (g and j). HeLa cells were treated with the SCAP inhibitor fatostatin at the indicated concentrations (0–5 µM) followed by ferroptosis induction using increasing concentrations of the GPX4 inhibitors RSL3 (100 nM, j) or ML210 (indicated concentration, g). **(k)** Dose-dependent sensitization of HeLa cells to RSL3-induced ferroptosis following treatment with the HMGCR inhibitor mevastatin (1 or 2 µM). In all experiments (g–k, and m), ferroptosis was suppressed by the lipophilic antioxidants ferrostatin-1 (Ferr, 1 µM) or liproxstatin-1 (Lip-1, 1 µM). **(l and m)** Generation and validation of *SCAP* KO HeLa cells. WT HeLa cells were transduced with lentiviral sgRNA against *SCAP* and selected with puromycin. **(l)** Quantification of total cholesterol levels via mass spectrometry in parental and *SCAP* KO polyclonal populations following 48 h of serum deprivation. **(m)** Real-time monitoring of cell death in *SCAP* KO cells using the Incucyte system. Cell death was quantified by normalizing the Sytox Green-positive area to the total phase-contrast area (*n* = 4). Error bars in a–m represent the 95% confidence interval (CI). P values are indicated above the respective data points. GPX4, glutathione peroxidase 4

The SCAP–SREBP axis activates the mevalonate pathway, which generates IPP to restore cholesterol levels (Fig. 1 e) (Horton et al., 2002). IPP also serves as a precursor for CoQ biosynthesis (Tran and Clarke, 2007). To understand why SCAP inhibition impairs FSP1- and CoQ-dependent ferroptosis resistance in cyto-STARD7 cells, we measured CoQ and cholesterol levels. While total CoQ levels remained unchanged, the levels of cholesterol esters significantly increased (Fig. 2, a and b). The abundance of the cholesterol 18:1 species more than doubled in these cells (Fig. 2 a). In contrast, other lipid classes, such as lyso-phosphatidylcholine, phosphatidylcholine (PC), lyso-phosphatidylethanolamine, phosphatidylethanolamine (PE), phosphatidylglycerol, diacylglycerol, free fatty acids, and sphingomyelin, showed no significant changes (Fig. S1, f–l). Since cholesterol esters accumulate as a storage form of excess cholesterol, these results suggest that activation of mevalonate pathway in cyto-STARD7 cells promotes cholesterol biosynthesis, whereas CoQ levels remained unaltered.

**Figure S2.**
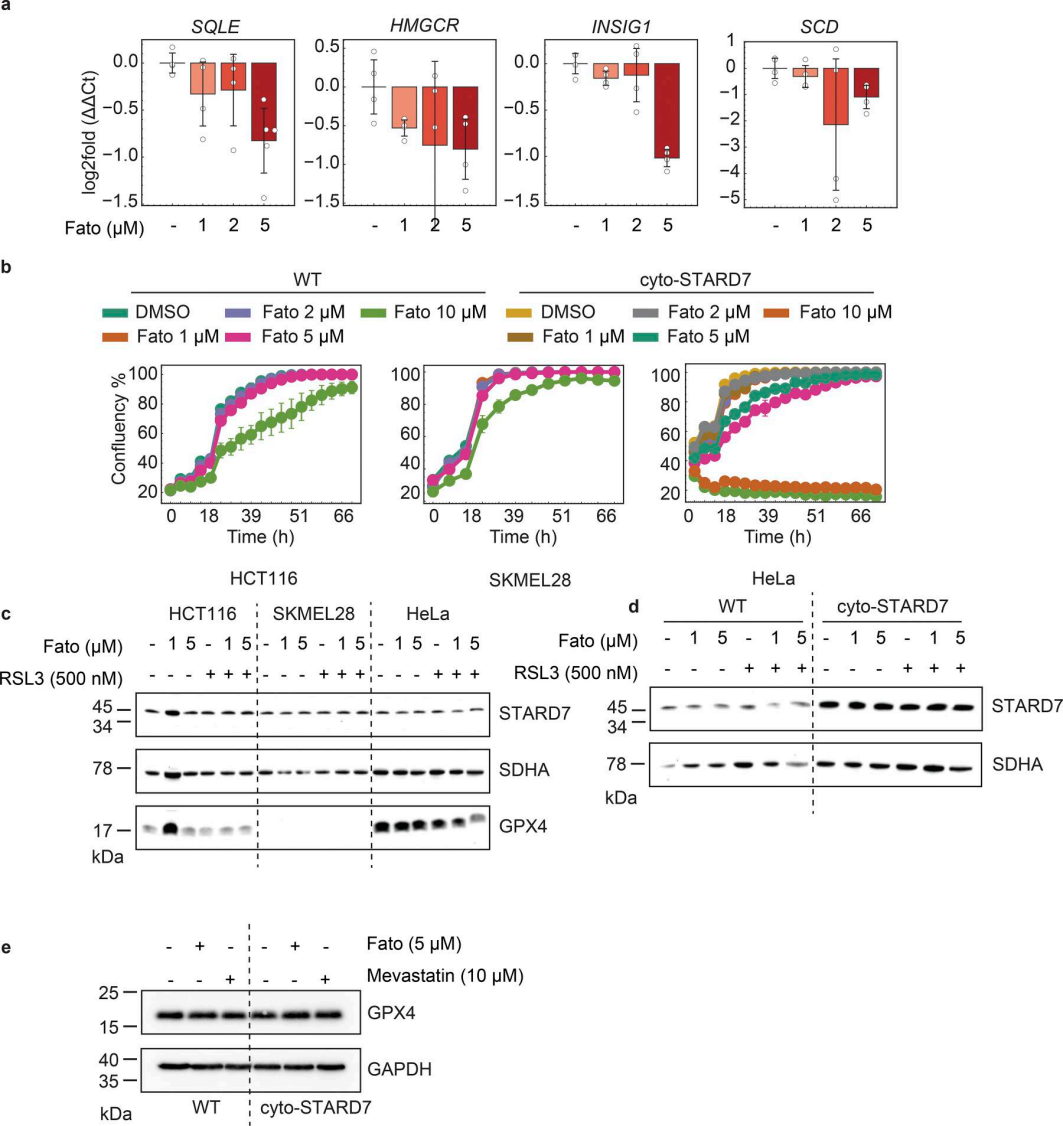
**Effect of SCAP inhibition on cell viability and GPX4 expression. (a)** WT HeLa cells were treated with the SCAP inhibitor fatostatin at the indicated concentrations for 24 h. Relative gene expression of SREBP-target genes, including SQLE, HMGCR, INSIG1, and SCD, was analyzed using the ΔΔCt method. Graphs represent LFCs in expression relative to the DMSO control (*n* = 5 biological replicates). **(b)** Cell growth was measured in HCT116, SKMEL28, and WT and cyto-STARD7 HeLa cells. Cells were treated with fatostatin in a dose-dependent manner to determine the nonlethal concentration of the drug. Cellular confluency was assessed using Incucyte phase contrast imaging (*n* = 3 biological replicates). **(c–e)** Western blot analysis of STARD7 and GPX4 protein expression in HCT116, SKMEL28, and WT and cyto-STARD7 HeLa cells. Cells were treated with either fatostatin or mevastatin at the indicated concentrations for 24 h, and ferroptosis was induced using the GPX4 inhibitor RSL3. Error bars in a and b represent the 95% confidence interval. SQLE, squalene epoxidase, INSIG1: insulin-induced gene 1, SCD, stearoyl-CoA desaturase; GPX4, glutathione peroxidase 4.Source data are available for this figure: SourceData FS2.

This finding was unexpected, as CoQ and cholesterol share upstream biosynthetic pathways (Fig. 1 e). To substantiate these findings, we pharmacologically inhibited the SCAP–SREBP pathway using fatostatin in both WT and cyto-STARD7 cells. As expected, the expression of canonical SREBP target genes decreased (Fig. S2 a). Within 24 h, the levels of cholesterol decreased, while those of CoQ remained unaffected (Fig. 2, c and d). A decrease in CoQ was observed only after 72 h (Fig. 2 e), suggesting that cholesterol levels are more sensitive to alterations in the mevalonate pathway. The different sensitivities of cholesterol and CoQ levels may be related to differences in the half-lives of the lipids. Therefore, we performed stable isotope tracing using ^13^C_6_-glucose. We treated cells with the mevalonate pathway inhibitors fatostatin, which inhibits SCAP, or 22(S)-hydroxycholesterol (22CR), which binds to INSIG and prevents SREBP from translocating to the nucleus. This was followed by ^13^C-glucose feeding of the cells for 5 or 24 h to trace the incorporation of the heavy carbon isotope into CoQ (Fig. 2 f). Neither treatment significantly reduced ^13^C-incorporation into CoQ after 5 h, we observed only a trend toward reduced CoQ synthesis after prolonged (48 h) fatostatin exposure. Collectively, these data show that cholesterol synthesis is sensitive to fluctuations in mevalonate pathway flux, whereas CoQ biosnythesis is more refractory.

To examine the relevance of these findings in different cell lines, we inhibited the SCAP–SREBP pathway in HCT116, SKMEL28, and HeLa cells and measured ferroptosis susceptibility. We first evaluated the dose-dependent effect of fatostatin on cell viability across the different cell lines and selected a non-lethal concentration (Fig. S2 b). Consistent with our CRISPR screen results, pharmacological SCAP inhibition sensitized HeLa cells to both RSL3- and ML210-induced ferroptosis within 24 h (Fig. 2, g–j). A similar sensitivity upon fatostatin treatment was observed in HCT116 and SK-MEL-28 cells (Fig. 2, h and i). Furthermore, SCAP inhibition effectively abrogated the ferroptosis resistance conferred by cyto-STARD7 expression, reinforcing the essential role of the SCAP–SREBP axis in mediating this protective phenotype (Fig. 2 j).

Given the pleiotropic effects of SCAP inhibition, we next investigated whether the specific inhibition of HMGCR, a key downstream target of the SCAP–SREBP pathway, would yield comparable results. Indeed, treatment with the HMGCR inhibitor mevastatin sensitized cells to RSL3-induced ferroptosis in a dose-dependent manner (Fig. 2 k). To rule out nonspecific pharmacological effects, we generated *SCAP*^*−/−*^ polyclonal HeLa cells by CRISPR genome editing. *SCAP* deletion resulted in significantly decreased cholesterol levels, confirming a functional blockade of the pathway (Fig. 2 l). Recapitulating the effects of fatostatin, *SCAP*^*−/−*^ cells exhibited increased sensitivity to ferroptosis induction, which was fully rescued by the lipophilic antioxidant liproxstatin-1 (Fig. 2 m). Notably, despite the marked change in sensitivity, neither *de novo* CoQ synthesis (as measured by isotope enrichment) nor total CoQ levels were altered after SCAP inhibition for 24 h (Fig. 2, d and f). These findings indicate that the increased ferroptotic sensitivity observed upon SCAP disruption is independent of alterations in total CoQ abundance.

### Inhibition of IPP production alters CoQ distribution

How does the loss of SCAP affect the ferroptotic resistance without affecting CoQ levels? To investigate whether SCAP inhibition affects GPX4 or STARD7 expression, potentially contributing to increased ferroptosis sensitivity, we assessed the protein levels of both STARD7 and GPX4 in HCT116, SKMEL28, and HeLa cells treated with the SCAP inhibitor fatostatin or the HMGCR inhibitor mevastatin, with or without ferroptosis induction. However, the expression of either protein was not altered under these conditions (Fig. S2, c–e).

**Figure S3.**
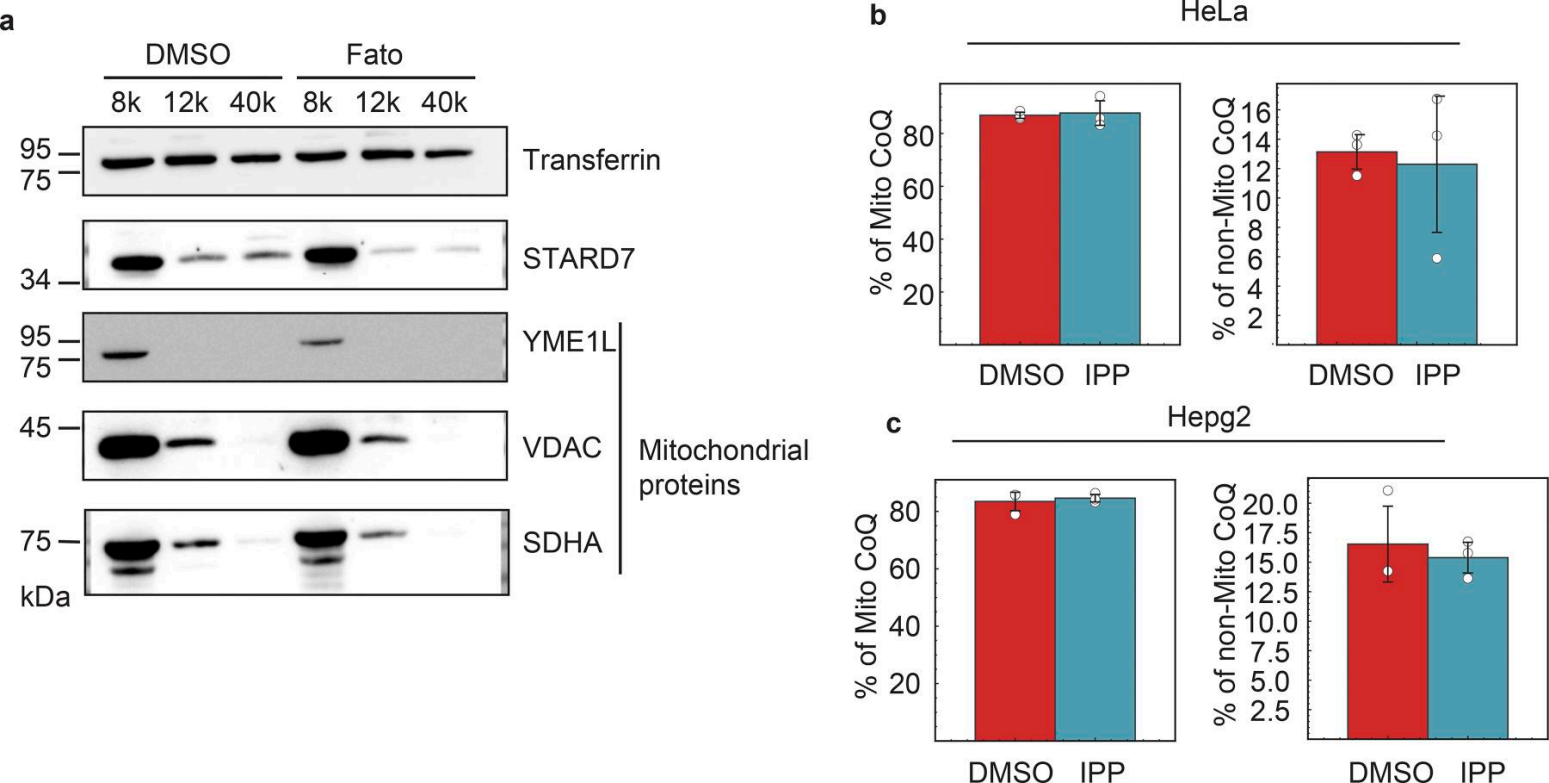
**CoQ distribution following SCAP inhibition and IPP supplementation. (a)** WT HeLa cells were treated with DMSO or fatostatin for 24 h, and mitochondria were extracted using differential centrifugation. The cells were centrifuged at different speeds (8,000*g*, 12,000*g*, and 40,000*g*) to separate mitochondrial fractions. Western blotting was performed to detect markers specific to mitochondrial (VDAC, SDHA, and YME1L) and PM (transferrin) fractions. **(b and c)** HeLa (b) and HepG2 (c) cells were treated with either DMSO or IPP for 24 h. Cells were collected, and mitochondrial and non-mitochondrial fractions were isolated using differential centrifugation (*n* = 3 biological replicates). The pellets from each fraction were sonicated in MTBE buffer, and lipids were extracted. The lipid phase was then analyzed by mass spectrometry to measure CoQ levels. MTBE, methyl-tert-butyl ether. Source data are available for this figure: SourceData FS3.

**Figure 3.**
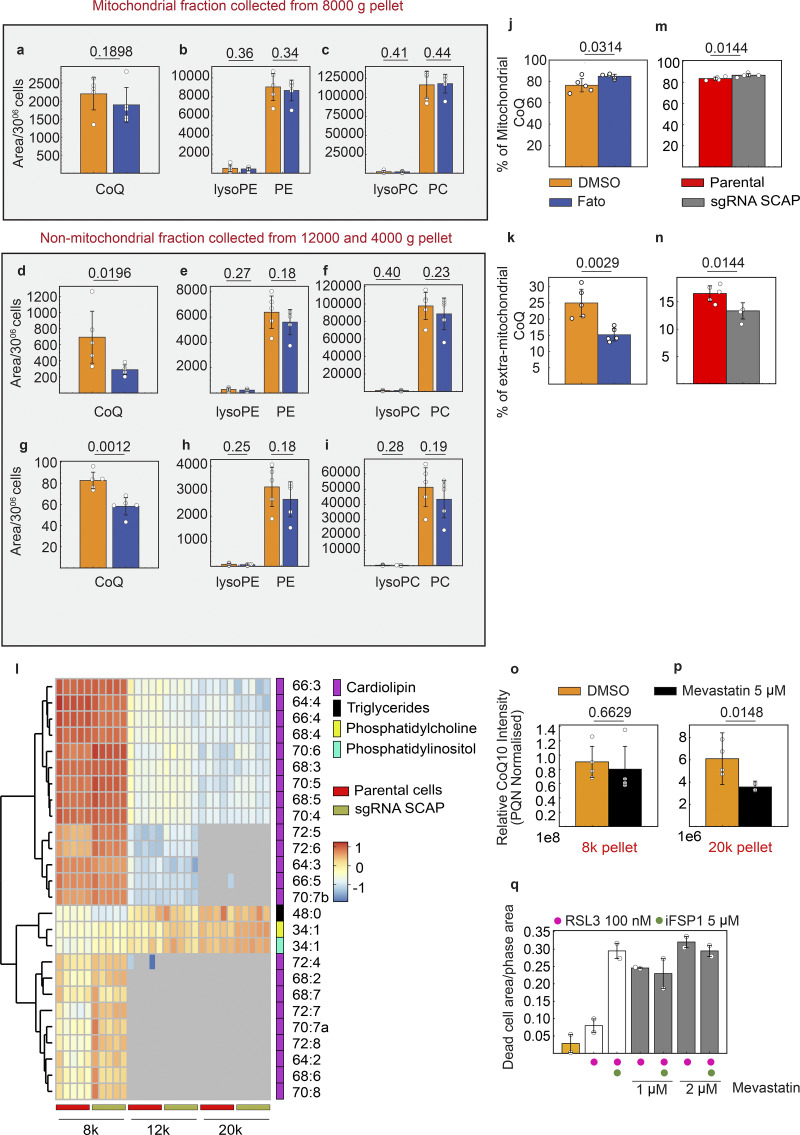
**CoQ distribution upon mevalonate pathway inhibition. (a–i)** HeLa cells (30 × 10^6^) were treated with DMSO or the SCAP inhibitor fatostatin (5 µM) for 24 h and used for the enrichment of mitochondria through differential centrifugation (*n* = 5). First, cells were centrifuged at 8,000 × *g* to pellet mitochondria, which were then sonicated in methyl-tert-butyl ether (MTBE) buffer (a–c). The supernatant was further centrifuged at 12,000 × *g* to isolate heavy membranes, followed by a 20,000 × *g* centrifugation to isolate non-mitochondrial membranes (d–i). Both pellets were resuspended in MTBE and sonicated for lipid extraction. The lipid phase was separated from the polar phase and analyzed for CoQ (a, d, and g), lysoPE and PE (b, e, and h), and lysoPC and PC (c, f, and i). **(j and k)** The percentage of mitochondrial CoQ (j) and extramitochondrial CoQ (k) was calculated in cells treated with either DMSO or fatostatin for 24 h. Total CoQ levels in cells are shown as the sum of peak areas from the 8,000 × *g*, 12,000 × *g*, and 20,000 × *g* pellets in each replicate. To calculate mitochondrial CoQ (j), the peak area from the 8,000 × *g* pellet was divided by total CoQ, and for extra-mitochondrial CoQ (k), the combined peak areas from the 12,000 × *g* and 20,000 × *g* pellets were divided by total CoQ. **(l–p)** Lipidomic profiling and CoQ quantification in parental and SCAP KO polyclonal HeLa cells. **(l)** Heatmap illustrating the subcellular distribution of CL species, TG, PC, and PI across the different fractions. The selective enrichment of CL in the 8k pellet validates the successful isolation of mitochondria. Gray cells in the heatmap represent lipids that were below the limit of detection. **(m and n)** CoQ levels in parental vs. *SCAP*^*−/−*^ cells and o and p, in WT HeLa cells treated with DMSO or the HMGCR inhibitor mevastatin for 48 h. **(q)** Epistasis analysis of mevalonate pathway inhibition and FSP1 activity. Real-time cell death monitoring in HeLa cells treated with mevastatin at indicated concentration (alone) or in combination with the FSP1 inhibitor iFSP1 (5 µM) following ferroptosis induction with RSL3. Cell death was quantified by normalizing the Sytox Green-positive area to the total phase-contrast area. Error bars in a–p represent the 95% confidence interval (CI). P values are indicated above the respective data points.

Since CoQ subcellular distribution critically influences ferroptotic sensitivity (Deshwal et al., 2023), we investigated whether the sensitivity observed upon SCAP inhibition was linked to defects in CoQ trafficking. We treated WT HeLa cells with DMSO or fatostatin and separated mitochondrial from other cellular membranes by differential centrifugation, a well-established method for monitoring CoQ distribution (Deshwal et al., 2023). The purity of the fractions was confirmed by the enrichment of mitochondrial markers (YME1L, VDAC, and SDHA) (Fig. S3 a). Mass spectrometry analysis revealed that CoQ levels in the mitochondrial fraction remained unaltered upon SCAP inhibition (Fig. 3 a), as did the levels of structural phospholipids, PE, and PC (Fig. 3, b and c).

In striking contrast, CoQ levels were significantly reduced in the non-mitochondrial fractions (Fig. 3, d and g), while PE and PC levels remained unaffected (Fig. 3, e, f, h, and i). Quantitative analysis of CoQ partitioning demonstrated that inhibition of the SCAP–SREBP pathway led to a significant decrease in non-mitochondrial CoQ, while the levels of mitochondrial CoQ showed a trend toward an increase (Fig. 3, j and k).

To strengthen these findings, we performed similar fractionation experiments using *SCAP*^*−/−*^ polyclonal HeLa cells. To rigorously validate our fractionation across genotypes, we analyzed a broad lipidomic profile, including cardiolipin (CL), triglycerides (TG), phosphatidylinositol (PI), and PC. Both parental and *SCAP*^*−/−*^ mitochondrial fractions showed a robust enrichment of 23 CL species and a relative decrease in TG, PC, and PI (Fig. 3 l). Conversely, CL was either heavily decreased or nearly undetectable in the 12k and 20k pellets, which were instead enriched with TG, PC, and PI. This lipidomic signature was highly reproducible across genotypes, confirming the precision of our membrane separation (Fig. 3 l). Consistent with our pharmacological data, *SCAP* deletion and specific HMGCR inhibition via mevastatin resulted in a depletion of extramitochondrial CoQ without altering mitochondrial CoQ levels (Fig. 3, m–p).

Finally, we sought to determine if the ferroptotic sensitivity induced by inhibition of mevalonate pathway is directly caused by an impaired CoQ export from mitochondria. We performed an epistasis analysis by co-inhibiting FSP1, which utilizes extramitochondrial CoQ to protect the PM. We reasoned that if HMGCR inhibition and FSP1 act within the same protective axis (CoQ availability at the PM), their combined inhibition should not be additive. Indeed, while mevastatin sensitized cells to RSL3-induced ferroptosis, the addition of the FSP1 inhibitor failed to further increase cell death (Fig. 3 q).

Collectively, these findings demonstrate that upon SCAP–SREBP inhibition and reduced HMGCR activity, mitochondria limit CoQ export and preferentially retain CoQ within mitochondrial membranes, effectively “stockpiling” their reserves at the expense of extra-mitochondrial redox defense.

### Increased CoQ export from the mitochondria depends on enhanced IPP production and cyto-STARD7

Since both SCAP and HMGCR inhibition resulted in decreased CoQ export from the mitochondria, we hypothesized that CoQ distribution is responsive to changes in the flux through the mevalonate pathway. IPP, a key intermediate in this pathway, is the primary mevalonate-derived metabolite that enters mitochondria to support CoQ biosynthesis. To determine whether IPP availability directly influences CoQ trafficking, we first supplemented HeLa and HepG2 cells with exogenous IPP. However, IPP treatment alone did not enhance CoQ export (Fig. S3, b and c), suggesting that IPP is not sufficient on its own to drive CoQ redistribution.

**Figure 4.**
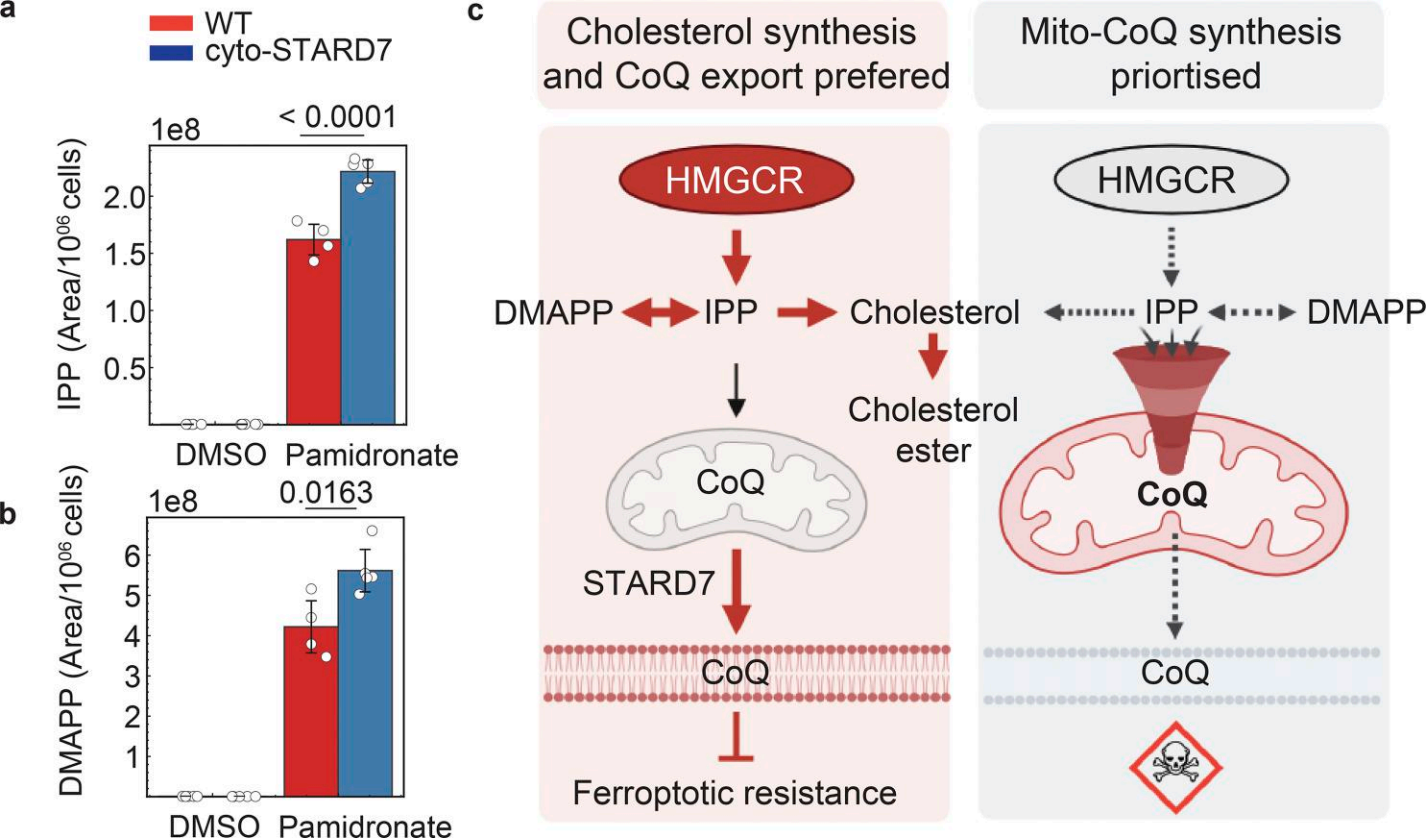
**CoQ distribution upon SCAP inhibition. (a and b)** WT and cyto-STARD7 HeLa cells were treated with DMSO or pamidronate (20 µM) for 8 h to inhibit farnesyl pyrophosphate synthase. For each replicate, 1 million cells were used to extract the polar phase using MTBE buffer. The polar phase was then analyzed for IPP (a) and dimethylallyl pyrophosphate (b) (*n* = 4). The error bars represent the 95% confidence interval. P values are shown above the graphs. **(c)** Schematic illustrating a potential mechanism by which mitochondria respond to isoprenoid precursor availability. Under conditions of high HMGCR activity and abundant IPP (e.g., cyto-STARD7 overexpression), the model suggests that cells preferentially channel IPP toward cholesterol biosynthesis, leading to cholesterol ester accumulation. This state correlates with enhanced mitochondrial CoQ export, which increases cytosolic CoQ levels and provides resistance to ferroptosis. Conversely, when IPP levels are limited (e.g., SCAP genetic or pharmacological inhibition or HMGCR inhibition), the data support a model where mitochondria prioritize endogenous CoQ synthesis over export, potentially as a survival mechanism that inadvertently sensitizes cells to ferroptosis. Created with BioRender.com.

We next asked whether endogenous IPP levels are elevated in cells with increased CoQ export, specifically, in cyto-STARD7 cells. However, mass spectrometry failed to detect IPP or its isomer dimethylallyl pyrophosphate in basal conditions (Fig. 4, a and b), likely due to their rapid turnover. To overcome this, we blocked a downstream step in the mevalonate pathway using pamidronate, an inhibitor of farnesyl diphosphate synthase, to promote IPP accumulation. After 8 h of treatment, pamidronate led to a detectable buildup of IPP, with cyto-STARD7 cells accumulating significantly more IPP than WT controls (Fig. 3, a and b). These results suggest that elevated cytosolic IPP levels, together with STARD7 overexpression, promote CoQ export from mitochondria.

In summary, our findings uncover a metabolic hierarchy, which prioritizes mitochondrial CoQ maintenance over cholesterol synthesis when the mevalonate pathway is compromised, whereas cholesterol synthesis is fostered when this pathway is upregulated. This protective mitochondrial state sustains respiratory function at the expense of mitochondrial CoQ export and ferroptotic resilience (Fig. 3 c). These results highlight the ability of mitochondria to dynamically regulate lipid partitioning across organelles, including the ER, to balance the competing demands of energy production and antioxidant defense.

The SCAP–SREBP axis is highly pleiotropic and several of its branches have been shown to influence ferroptosis. For instance, metabolic intermediates such as 7-dehydrocholesterol and squalene modulate ferroptotic sensitivity (Freitas et al., 2024; Li et al., 2024; Garcia-Bermudez et al., 2019; Zhang et al., 2024; Yamada et al., 2024), while SCD, a key SREBP-1 target, dictates the MUFA-to-PUFA ratio to suppress lipid peroxidation (Sen et al., 2023). While *SCD1* emerged in our CRISPR screen, we observed no significant alterations in MUFA-to-PUFA ratios in cyto-STARD7 cells (Fig. S1 l). Thus, our data identify mitochondrial CoQ distribution as a novel metabolic link between the mevalonate pathway and ferroptosis. We propose that ferroptosis resistance is regulated at multiple nodes within the mevalonate pathway, emphasizing a coordinated control system that preserves redox balance, respiration, and membrane integrity.

Our results demonstrate that neither the upregulation nor the inhibition of the mevalonate pathway affects total CoQ levels or ^13^C_6_-glucose incorporation into CoQ within 48 h, indicating that CoQ biosynthesis in the mitochondria is preserved. Cells may regulate this process by altering the uptake of IPP between mitochondria and the ER. In cyto-STARD7 cells, where HMGCR is upregulated and more IPP is available, cholesterol esters increase, but CoQ levels remain unchanged, suggesting that IPP is preferentially directed toward cholesterol synthesis under these conditions. Conversely, when SCAP is inhibited, cholesterol levels decrease while CoQ levels remain stable, probably by increasing IPP flux into the mitochondria and/or decreased CoQ efflux from mitochondria (Fig. 3 c).

Indeed, our data show that increased IPP availability promotes CoQ export in cyto-STARD7 cells, whereas inhibition of IPP synthesis reduces export, indicating that CoQ trafficking is sensitive to mevalonate pathway activity. Due to its hydrophobicity, CoQ export may depend on mitochondrial membrane remodeling processes such as mitochondria-derived vesicles or mitochondria-derived compartments, or it may occur at contact sites between mitochondria and the ER (Picca et al., 2023; Neuspiel et al., 2008; Wilson et al., 2024).

Unlike CoQ, which is produced almost entirely endogenously, cholesterol can be absorbed from the diet, influencing its synthesis through feedback inhibition (Duan et al., 2022; Brown and Goldstein, 1986; Siperstein and Fagan, 1966). This adds another layer of regulation to the mevalonate pathway. Therefore, our discovery of a metabolic hierarchy between cholesterol and CoQ synthesis raises the intriguing possibility that dietary cholesterol may alter the levels or cellular distribution of endogenous CoQ, especially under conditions of metabolic challenge.

The regulation of CoQ and cholesterol occurs in a tissue-specific manner. Cholesterol is predominantly synthesized in the liver, whereas mitochondrial CoQ biosynthesis occurs in all cells (Duan et al., 2022; Tran and Clarke, 2007). The synthesis of these lipids is responsive to the metabolic state. In obesity, for example, hepatic CoQ biosynthesis is impaired while cholesterol levels remain unchanged, pointing to a failure to reprioritize CoQ production under metabolic stress (Goncalves et al., 2025).

Together, our findings highlight how mitochondria dynamically allocate metabolic resources to prioritize respiratory function. Understanding how this regulation breaks down in disease may open new avenues for targeting ferroptosis and metabolic dysfunction.

## Materials and methods

### Cell culture

HeLa (CCL-2) cells were obtained from the American Type Culture Collection (ATCC) and PARL^−/−^, cyto-STARD7 HeLa cells were generated in the lab (Deshwal et al., 2023). The human colorectal carcinoma HCT116 cell line (catalog number CCL-247) was sourced from ATCC. SKMEL28 cells were kindly provided by Paola Zigrino’s lab, and HepG2 cells were a gift from Lena Pernas’s lab. Cells were cultured in DMEM supplemented with GlutaMAX (Life Technologies) and 10% FBS at 37°C in a 5% CO_2_ environment. For metabolomics flux experiments, DMEM without glucose was used, supplemented with ^13^C_6_ glucose. All cell lines were routinely tested for *Mycoplasma* contamination. For drug treatments, cells were plated for 24 h and subsequently treated with various compounds at the indicated concentrations. The compounds used included RSL3 (SML2234), ferrostatin-1 (SML 0583), fatostatin (341329), 22(S)-hydroxycholesterol (22CR) (H5884), all purchased from Sigma-Aldrich.

### CRISPR screen

#### CRISPR KO fitness screen

The genome-wide VBC-score library, containing 124,604 sgRNAs (five sgRNAs per gene, including 600 nontargeting control sgRNAs), was a gift from Uli Elling (ViVerita Discovery, Vienna, Austria) (Michlits et al., 2020). Lentiviral particles were produced by transfecting the library plasmid along with lentiviral packaging plasmids (psPAX2 and pMD2.G) into HEK293T cells using Lipofectamine 3000 (L3000015; Invitrogen). The plasmids psPAX2 and pMD2.G were gifts from Didier Trono (École Polytechnique Fédérale de Lausanne [EPFL], Lausanne, Switzerland) (#12260 and #12259; Addgene plasmids). Lentiviral supernatants were collected 48 h after transfection, filtered (0.45 µm), and stored at −80°C. The optimal lentiviral volume for the screen was determined by transduction of 3.5 × 10^6^ HeLa cells with a range of viral volumes for overnight in the presence of 6 µg/ml Polybrene (sc-134420; Santa Cruz). After 48 h, cells were split into selection medium with or without 500 µg/ml G418 (#10131035; Thermo Fisher Scientific). The optimal virus volume resulted in ∼30% survival after G418 selection (MOI of ∼0.3).

The CRISPR screen was conducted in Cas9-expressing cyto-STARD7 HeLa cells with a screen coverage of >300 ×. 140 × 10^6^ cells were transduced with the lentiviral library at an MOI of ∼0.3. Selection with 500 µg/ml G418 was performed, with uninfected cells subjected to the same conditions in parallel. After selection, cells were split into DMSO and ferroptosis inducer RSL3 (100 nM) treatment groups in technical duplicates. Cells were passaged every 3–4 days, maintaining a minimum of 300 × coverage (80 × 10^6^ cells). The screen continued for 14 days or 12 population doublings, with cell pellets harvested at the T12 endpoint.

Genomic DNA (gDNA) was extracted using the QIAamp DNA Blood Maxi Kit (#51194; Qiagen) according to the manufacturer’s instructions. gRNA sequences were amplified from gDNA by PCR (24 cycles using 5 µg of gDNA as input) with Q5 High-Fidelity DNA polymerase (M0544X; NEB) and a mix of forward primers (NGS-F1 to NGS-F1.5) and a reverse primer (NGS-R4a) (Table 1). PCR amplicons were pooled, bead-purified, quantified, and subjected to a second round of PCR to introduce Illumina Nextera adaptors and indices. The final library was bead-purified, quantified, pooled, and analyzed by paired-end (2 × 100 bp) sequencing on an Illumina NovaSeq platform, with >36 × 10^6^ reads per sample.

**Table 1.** List of primers used for PCR1 to amplify guide RNA sequences from genomic DNA

Primer	Sequence
CRISPR NGS F1	5′-TCG​TCG​GCA​GCG​TCA​GAT​GTG​TAT​AAG​AGA​CAG​TGT​GGA​AAG​GAC​GAA​ACA​CC-3′
CRISPR NGS F1.1	5′-TCG​TCG​GCA​GCG​TCA​GAT​GTG​TAT​AAG​AGA​CAG​NTG​TGG​AAA​GGA​CGA​AAC​ACC-3′
CRISPR NGS F1.2	5′-TCG​TCG​GCA​GCG​TCA​GAT​GTG​TAT​AAG​AGA​CAG​NNT​GTG​GAA​AGG​ACG​AAA​CAC​C-3′
CRISPR NGS F1.3	5′-TCG​TCG​GCA​GCG​TCA​GAT​GTG​TAT​AAG​AGA​CAG​NNN​TGT​GGA​AAG​GAC​GAA​ACA​CC-3′
CRISPR NGS F1.4	5′-TCG​TCG​GCA​GCG​TCA​GAT​GTG​TAT​AAG​AGA​CAG​NNN​NTG​TGG​AAA​GGA​CGA​AAC​ACC-3′
CRISPR NGS F1.5	5′-TCG​TCG​GCA​GCG​TCA​GAT​GTG​TAT​AAG​AGA​CAG​NNN​NNT​GTG​GAA​AGG​ACG​AAA​CAC​C-3′
CRISPR NGS R4a	5′-GTC​TCG​TGG​GCT​CGG​AGA​TGT​GTA​TAA​GAG​ACA​GCT​AGA​GCC​ATT​TGT​CTG​CAG​A-3′

### CRISPR KO live–dead screen

After selection with G418 and expansion of stably transduced cell pools, cells were treated with DMSO or RSL3 (500 nM) for 48 h in technical duplicates. Cells were washed with PBS, trypsinized, and centrifuged at 2500 g for 5 min. The cell pellets were resuspended in PBS, and cell concentrations were adjusted to 1 million of cells per mL. Prior to fixation, cells were stained with a fixable live–dead marker (LIVE/DEAD Fixable Green Kit, L34970; Thermo Fisher Scientific) for 30 min at a 1 μl/ml concentration on ice. Cells were then fixed in 2% paraformaldehyde (PFA) for 15 min in 2% FBS in 1 × FACS buffer (PBS with 1% BSA), followed by a brief spin at 300 g for 5 min and washed in FACS buffer to remove the fixative. ∼8 × 10^6^ cells per replicate were sorted into live and live–dead stain–positive populations using 488 nm excitation on two cell sorters in parallel (BD FACSAria III and BD FACSFusion). Cell pellets were stored at −80°C. Presort pellets, with >300 × coverage, were also harvested.

Before gDNA extraction, fixed cell pellets were de–cross-linked in ATL buffer (19076; Qiagen) with Proteinase K (19133; Qiagen) at 55°C for 1 h, followed by incubation at 90°C for 30 min. After de–cross-linking, gDNA extraction was performed as described above.

### CRISPR KO screen data analysis

After demultiplexing, raw NGS libraries were quality-checked using FastQC version 0.11.8 (Babraham Institute, Cambridge, UK. FastQC: A quality control tool for high throughput sequence data). Upstream sequences and gRNA length was used to trim reads with cutadapt (version 4.5). MAGeCK (version 0.5.9.5) was used to quantify the number of reads per gRNA (Li et al., 2014). Raw gRNA counts were median-normalized, and MAGeCK test was used to rank gRNAs. The log2-fold change (LFC) at the gene level was calculated as the median of the LFCs at the gRNA level. For gene significance, an α-RRA score and FDR were calculated by MAGeCK (Li et al., 2014) and plotted as a double-sided volcano plot. All genes with FDR <0.05 are highlighted with red circles, and the list of all significant genes is provided in the table (Fig. S1, d and e).

### Generation of SCAP-KO cells using the CRISPR–Cas9 system

The lentiCRISPRv2 vector capable of producing high-titer virus was used to generate KO cells (#52961; Addgene plasmid). The following sgRNA targeting the SCAP gene was cloned into the lentiCRISPRv2 vector and verified by DNA sequencing: 5′-TCT​CAC​GCA​GCC​TTT​CAG​TC-3′ (Uota et al., 2024). To produce lentivirus, ∼24 h before transfection, 4–5 × 10^6^ HEK293T cells were seeded in 10-cm plates in 8 ml of growth medium, using the Lenti-X Packaging Single Shots (VSV-G) (Cat. No. 631275), according to the manufacturer’s instructions. The supernatant containing lentivirus was harvested from the transfected cells, centrifuged, and stored at −80°C until use. For lentiviral transduction, HeLa cells (3 × 10^5^) were seeded in 6-well plates, and the virus was added to the cells, including polybrene at 8 μg/ml final concentration. The cells were cultured for 24 h, followed by the addition of fresh DMEM supplemented with 10% FBS, and treated for 5 days with 2 μg/ml puromycin for selection. The puromycin-resistant cells were then expanded in regular culture medium. Polyclone KO cells were validated via sequencing and lipidomics.

### Lipids extraction and measurements

For metabolite extraction, cells were processed using a biphasic methyl-tert-butyl ether–based method. 900 μl of cold lipid extraction buffer was pre-aliquoted into 2-ml Eppendorf tubes and kept on ice. Cells were incubated with 400 μl of pre-cooled (−20°C) initial extraction buffer (60% UPLC-MS grade MeOH in UPLC-MS grade water) for 10 min at −20°C. After incubation, cells were scraped and transferred into the corresponding extraction tubes, with an additional 400 μl of initial extraction buffer added to recover remaining material from each 6-well. Samples were incubated on a thermomixer at 4°C and 1,500 rpm for 30 min, followed by centrifugation at 21,000 *g* for 10 min at 4°C. An aliquot from the cleared supernatant was removed for cholesterol and fatty acid analysis. The remaining supernatant was transferred to new 2-ml Eppendorf tubes containing 200 μl of UPLC–MS grade water, while the protein pellet was retained for BCA. After a 10-min incubation at 15°C and 1,500 rpm, samples were centrifuged at 5,000 g for 5 min to allow phase separation. The upper (lipid) phase was collected (for CoQ and cholesterol ester measurement). CoQ levels were measured as previously described (Deshwal et al., 2023). For cholesterol and fatty acid measurements, lipid extracts were derivatized using a two-step process: first, methoxyamination with a 40 mg/ml methoxyamine solution in pyridine for 45 min at 40°C, followed by trimethylsilylation with MSTFA for an additional 45 min at 40°C. After derivatization, samples were centrifuged, and the supernatant was transferred to fresh autosampler vials. GC-MS analysis was performed by injecting 0.5 μl of the derivatized sample using a TriPlus RSH autosampler in splitless mode at 250°C, with helium as the carrier gas at 1 ml/min. Separation occurred on a 30 m MEGA-5 MS capillary column (0.25 mm diameter, 0.25 µm film thickness), with a temperature program of 1 min at 70°C, followed by a 9°C/min ramp to 350°C, and a final hold at 350°C for 5 min. The transfer line and source temperatures were both set to 280°C, and the mass spectrometer operated in full scan mode (m/z 70–700) with a scan speed of 20 Hertz. Data analysis for all compounds was performed using TraceFinder software, with compound identity validated by comparing retention indices and electron ionization spectra with authentic reference compounds. Extracted ion chromatograms were generated with a mass accuracy of <5 ppm and a retention time tolerance of <0.05 min.

### Western blotting

After treatment, cells were lysed with RIPA buffer (25 mM Tris-HCl [pH 8], 0.1% SDS, 150 mM NaCl, and 1% NP-40), supplemented with protease and phosphatase inhibitor cocktail (Cat. 78440; Thermo Fisher Scientific) and collected in 1.5-ml tubes. Lysates were quantified using the Pierce BCA Protein Assay Kit (Cat. 23227; Thermo Fisher Scientific), and, according to protein abundance, 20–40 μg of protein were mixed with the appropriate amount of Laemmli Sample Buffer 4X (Bio-Rad). Samples were then boiled at 95°C for 5 min, separated by SDS-PAGE, and transferred to a nitrocellulose membrane using the TransBlot Turbo TM (Bio-Rad) according to the manufacturer’s instructions. The membranes were blocked in 5% skim milk (Cat. 84615.05; VWR) for 60 min at room temperature. Subsequently, membranes were incubated with the indicated primary antibody overnight at 4°C. The appropriate secondary antibodies conjugated to HRP were used, and detection was carried out using ChemiDocTM Imaging System (Bio-Rad).

### Cell death assays

Cell death was measured using an Incucyte Live-Cell Analysis system (Sartorius) (Deshwal et al., 2023). Briefly, 5,000 cells were seeded per well in a 96-well plate. The following day, the medium was replaced with fresh medium, and cells were treated with the indicated compounds in the presence of 150 nM Sytox Green nucleic acid stain (S7020; Thermo Fisher Scientific). Phase-contrast and green fluorescence images were captured every 3 h. To quantify cell death, a mask was generated using the Incucyte base analysis software, analyzing both phase-contrast and green channels. The area occupied by green objects was normalized to the phase area per well.

### Isolation of mitochondrial and PM fractions

Mitochondrial and PM fractions were separated from cells using differential centrifugation (Deshwal et al., 2023). Briefly, cells were homogenized in an isolation buffer containing 20 mM HEPES/KOH (pH 7.4), 220 mM mannitol, 70 mM sucrose, and 1 mM EGTA using a Potter homogenizer at 1,000 rpm. The whole-cell sample was collected following homogenization. The homogenate was first centrifuged at 8,000 × *g* for 10 min at 4°C to isolate mitochondria. The resulting supernatant was then centrifuged again at 8,000 × *g* for 10 min at 4°C to collect additional mitochondrial material. The 8,000 × *g* pellets were pooled to form the mitochondrial fraction. To remove any remaining mitochondrial contamination, the supernatant was centrifuged at 12,000 × *g* for 15 min at 4°C. For PM isolation, the supernatant was subjected to two rounds of centrifugation: first at 20,000 × *g* for 45 min at 4°C, followed by centrifugation at 100,000×*g* for 1 h at 4°C. The following samples were collected for mass spectrometry analysis: 8,000 × *g*, 12,000 × *g*, 20,000 × *g*.

### Quantitative real-time PCR

mRNA was isolated from HeLa cells using the NucleoSpin RNA kit (Macherey-Nagel; 740,955.250). cDNA synthesis was carried out with the Promega GoScript Reverse Transcriptase kit (Cat# A5003) and adjusted to a final concentration of 10 μg/ml. TaqMan Gene Expression Master Mix (Applied Biosystems) was used for PCR amplification, with 0.6 μM of each primer and 10 ng of cDNA. The reaction was carried out over 30 cycles, consisting of a 95°C melting step and a 60°C elongation step. HPRT and GAPDH were used as housekeeping genes for normalization. Gene expression was calculated using the LFC (ΔΔCt) method.

### Statistics and reproducibility

All graphs represent data from independent experiments or biological samples. Datasets were analyzed using Instant Clue software version 3.0. Data are presented as the 95% confidence interval of the mean, highlighting statistically significant differences between groups. Statistical significance was determined with a two-tailed Student’s *t* test for unpaired comparisons, with a threshold of P < 0.05. Sample size was not predetermined using statistical methods, and no data were excluded from the analyses. Investigators conducting proteomics and metabolomics measurements were blinded to sample allocation, and samples were randomized. For all other experiments, investigators were not blinded, and samples were not randomized.

### Antibodies

The following commercial antibodies were used STARD7 (15689-1-AP; Proteintech, dilution 1:2,500), SDHA (Ab14715; Abcam, dilution 1:10,000), YME1L (11510-1-AP; Proteintech, dilution 1:1,000), VDAC2 (11663-1-AP; Proteintech, dilution 1:1,000), Transferrin (13-6800; Invitrogen, dilution 1:2,000), and GPX4 (125066; Abcam, dilution 1:1,000. All primary antibodies were diluted in 5% milk-TBST, and nitrocellulose membranes were incubated overnight at 4°C.

HRP-conjugated secondary antibodies used were goat anti-rabbit (170-6515; Bio-Rad, dilution 1:5,000) and goat anti-mouse (170-6516; Bio-Rad, dilution 1:5,000). For GPX4 detection, anti-rabbit IgG, HRP-linked antibody (#7074S; Cell Signaling Technology, dilution 1:3,300) was used. All secondary antibodies were diluted in 5% milk-TBST and incubated for 1 h at room temperature.

### Online supplemental material

Fig. S1 demonstrates the functional validation of Cas9 expression and comprehensive lipid profiling in cyto-STARD7 cells, Fig. S2 illustrates the effect of SCAP inhibition on cell viability and GPX4 expression, and Fig. S3 shows CoQ distribution following SCAP inhibition and IPP supplementation.

## Supplementary Material

SourceData FS2 is the source file for Fig. S2.

SourceData FS3 is the source file for Fig. S3.

## Data Availability

The data underlying Figs. 1, 2, 3, and 4 are available in the published article and its online supplemental material.
