# Supplementary figures and images for: Mitochondria limit coenzyme Q export under cholesterol biosynthetic stress
Source: J Cell Biol. 2026 Jun 8;225(8):e202507174. doi: 10.1083/jcb.202507174 (PMC13245278; doi:10.1083/jcb.202507174)

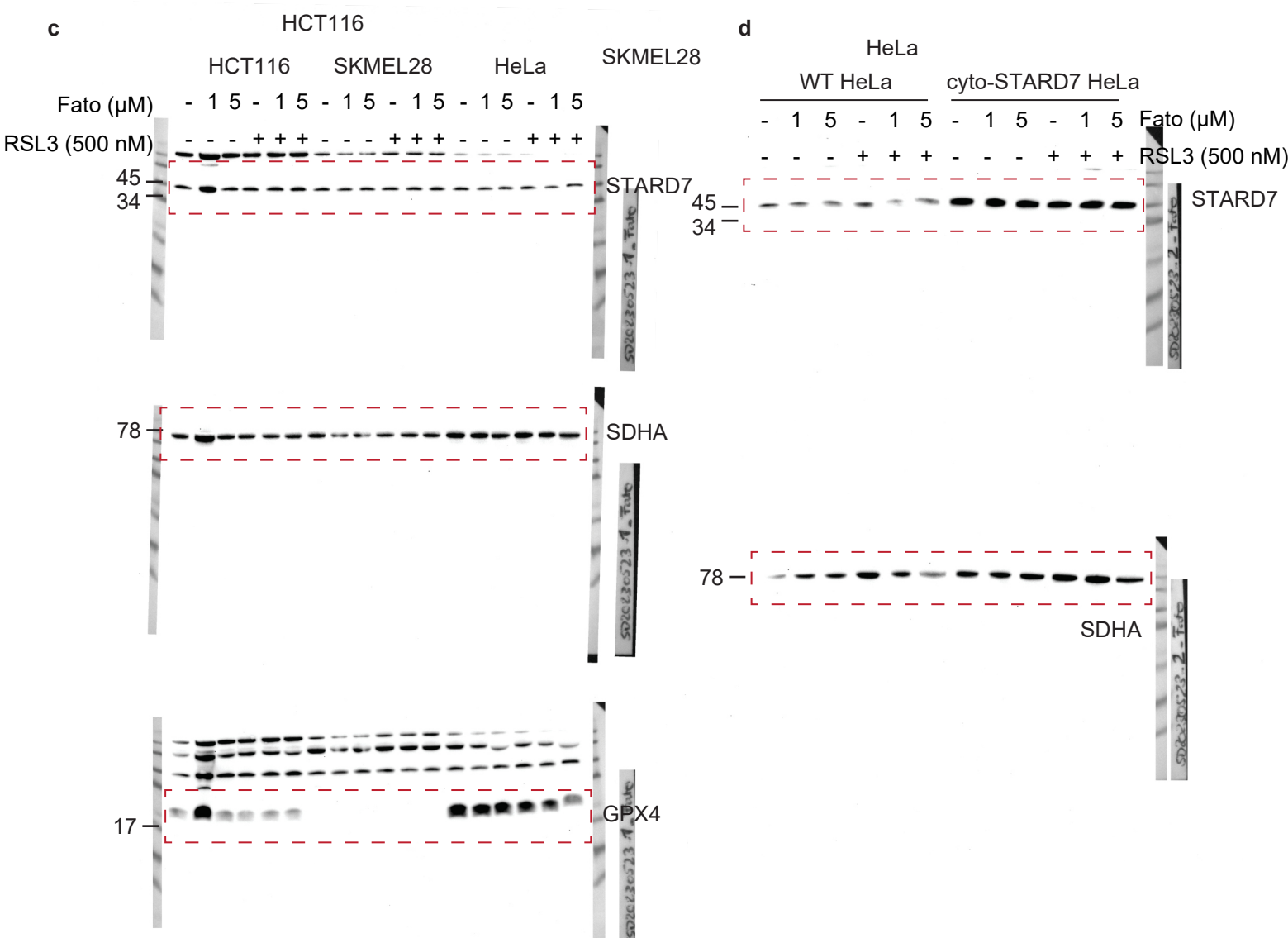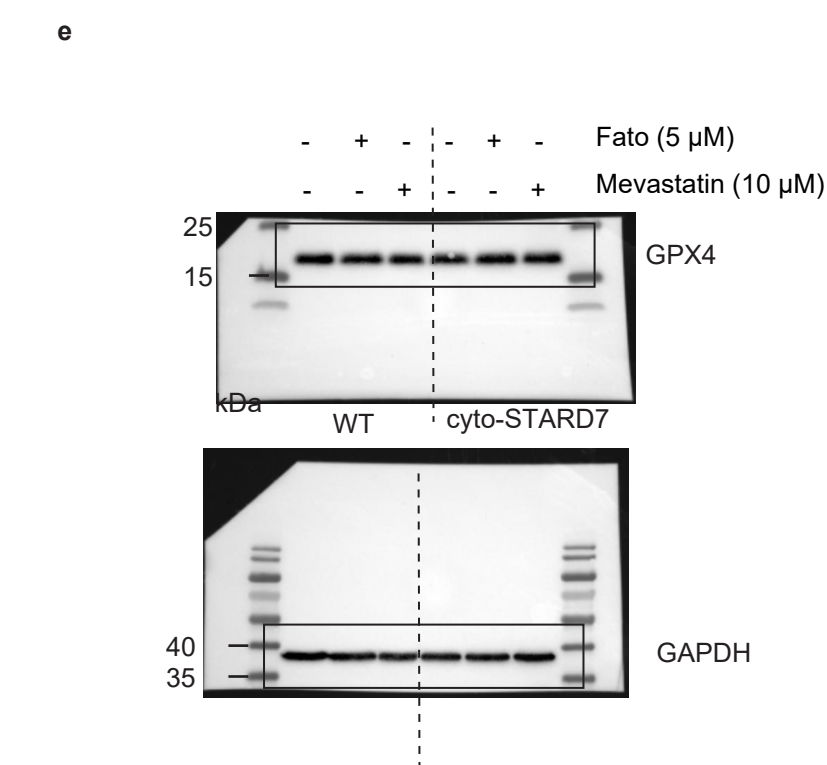

Supplement: SourceData FS2 — is the source file for Fig. S2. [file jcb_202507174_sourcedatafs2.pdf]

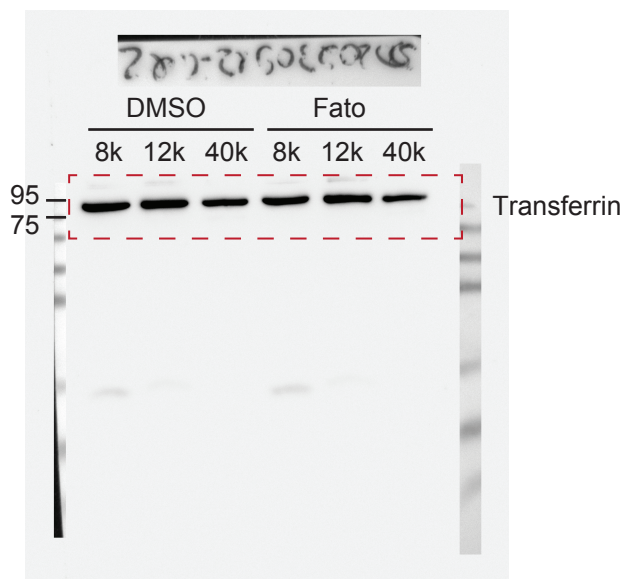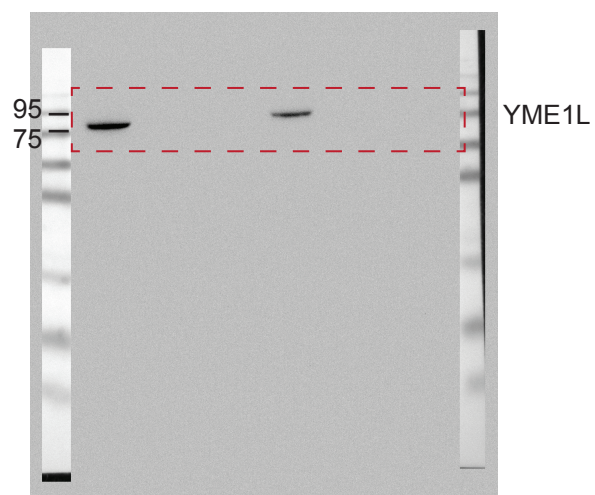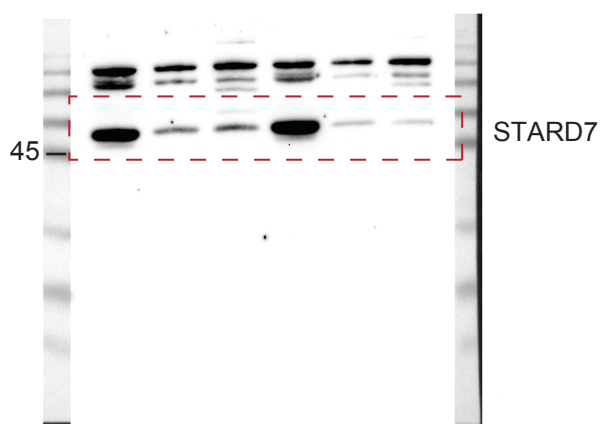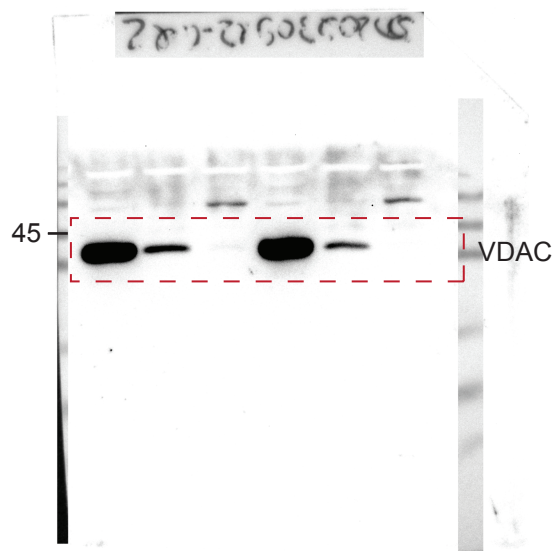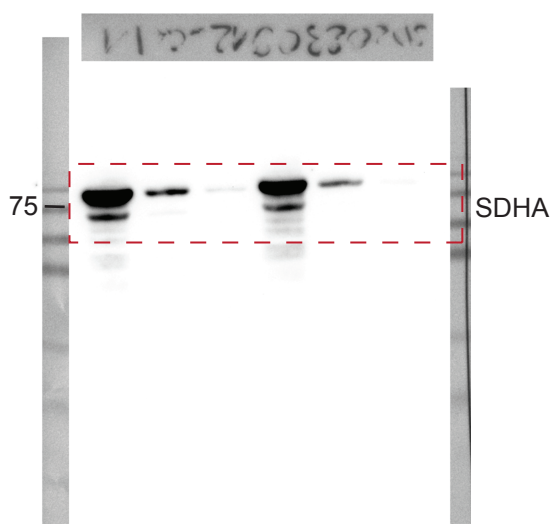

Supplement: SourceData FS3 — is the source file for Fig. S3. [file jcb_202507174_sourcedatafs3.pdf]
